# Primary Hepatocellular Carcinoma Developing Within a B-cell Primary Hepatic Lymphoma in a Hepatitis C Positive Patient

**DOI:** 10.7759/cureus.88274

**Published:** 2025-07-18

**Authors:** Tushar Kumar, Harmanjit Kaur, Surbhi Singh, Lakshmisree A Vemulakonda, Rajat Bhargava

**Affiliations:** 1 Department of Radiology, University of Washington School of Medicine, Seattle, USA; 2 Department of Internal Medicine, Boston Medical Center, Brighton, USA; 3 Department of Hematology and Oncology, The Brooklyn Hospital Center, Brooklyn, USA; 4 Department of Pathology, University of Washington School of Medicine, Seattle, USA

**Keywords:** hepatitis c, hepatocellular carcinoma, histopathological diagnosis, imaging features, primary hepatic lymphoma, primary hepatocellular carcinoma

## Abstract

Hepatocellular carcinoma is the most prevalent form of primary liver cancer, commonly associated with chronic liver conditions like hepatitis and alcohol abuse. Primary hepatic lymphoma (PHL), a rare type of non-Hodgkin lymphoma originating in the liver, primarily affects men around the age of 50. Chronic hepatitis B and C are also recognized as risk factors for PHL. Although there are a few case reports of hepatocellular carcinoma and PHL developing simultaneously in different areas of the liver, this report highlights a unique case where hepatocellular carcinoma developed within a previously treated B-cell hepatic lymphoma, an extremely uncommon situation. This rare occurrence may be due to factors like chronic inflammation, liver disease, and the effects of treatment. MRI and CT scans are the main diagnostic methods, and follow-up imaging demonstrating a different imaging pattern within the tumor compared to the baseline imaging should prompt a tissue diagnosis with histopathological analysis to identify a new tumor subtype, or a new tumor altogether, as in our case. A multidisciplinary approach is essential for managing such cases, and close monitoring is vital as underlying liver disease and multiple cancers can greatly impact prognosis. Despite medical advancements, the prognosis for advanced hepatocellular carcinoma remains bleak, with a 5-year survival rate under 5% in advanced stages.

## Introduction

Hepatocellular carcinoma is the most prevalent form of primary liver cancer, representing around 90% of cases. The global incidence of hepatocellular carcinoma has been rising, with an estimated 372,000 new diagnoses each year. Its development is frequently linked to underlying liver conditions like chronic viral hepatitis, excessive alcohol consumption, and non-alcoholic fatty liver disease. Individuals with chronic hepatitis C face a higher risk of developing cirrhosis, a key factor that increases the likelihood of developing hepatocellular carcinoma [1,2].

Primary hepatic lymphoma (PHL) is a rare form of extra-nodal non-Hodgkin lymphoma that originates in the liver. Although PHL makes up only 0.4% of all non-Hodgkin lymphomas and less than 1% of all liver cancers, the B-cell subtype is the most common, accounting for roughly 70% of cases, according to existing studies [1,3,4]. PHL primarily affects men, with a typical age of 50 years. While there have been a few case reports describing the development of hepatocellular carcinoma along with a B-cell hepatic lymphoma patient who is hepatitis C-positive, the simultaneous occurrence of these two distinct cancers in the same patient at the same, exact location is considered extremely rare [2,5].

## Case presentation

**Figure 1:**
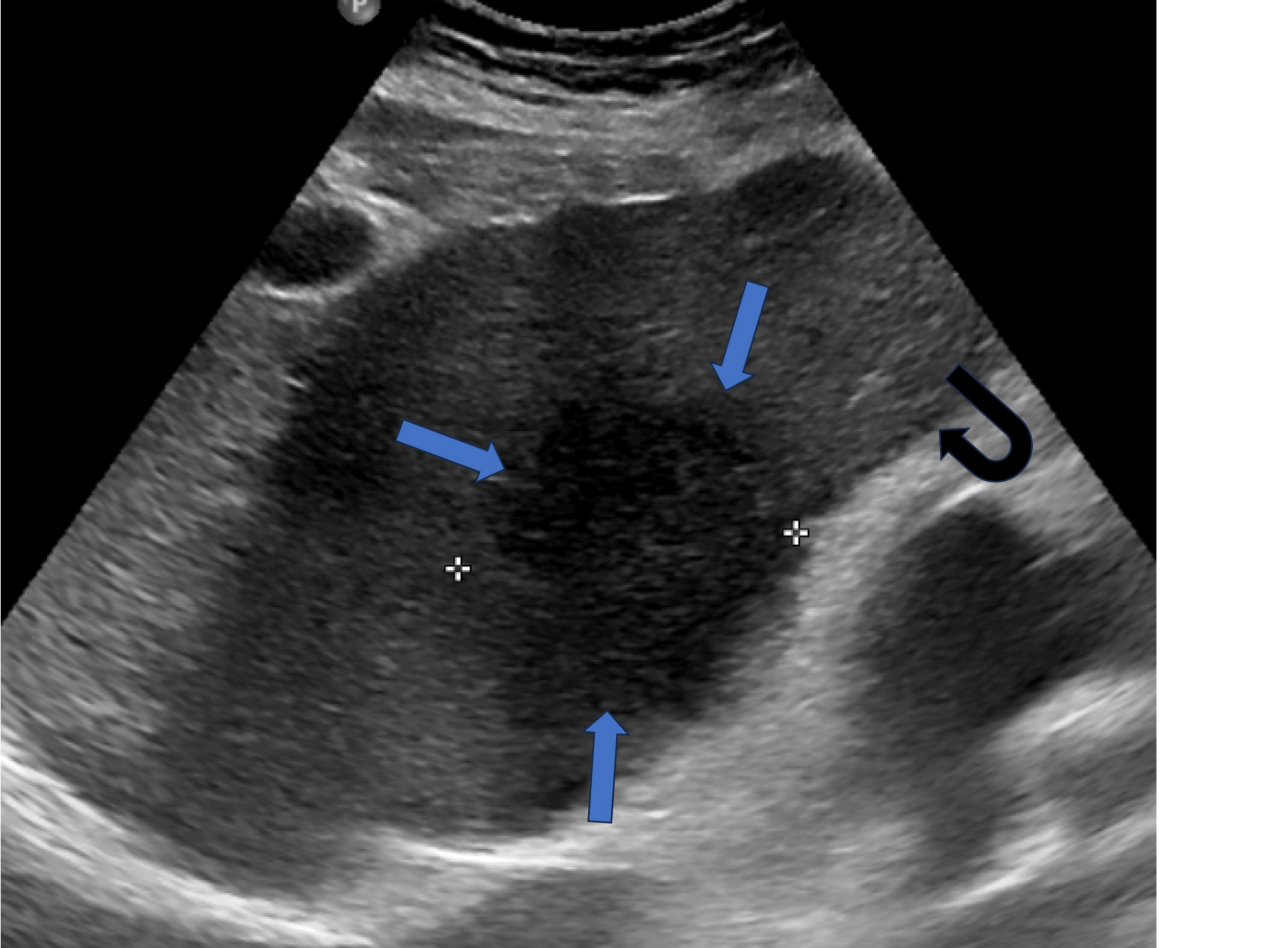
Transabdominal ultrasound of the abdomen demonstrating a hypoechoic lesion in the left lobe (blue arrows), suspicious of a malignancy The nodular liver margins represent cirrhosis (black curved arrow).

A 70-year-old male with a past history significant for controlled type 2 diabetes mellitus, smoking, consumption of alcohol, and hepatitis C presented with weakness and fatigue. He had completed antiviral treatment for hepatitis C with simeprevir a few years back and was cured. Family history was not significant for any concerning disease. Routine bloodwork (Table 1) demonstrated iron deficiency anemia, and esophagogastroduodenoscopy revealed varices and portal gastropathy. He underwent a non-emergent ultrasound to look for signs of cirrhosis and portal hypertension, which showed a suspicious lesion in the liver in addition to features of cirrhosis (Figure 1).

**Table 1:** Routine bloodwork of the patient demonstrating anemia Low hemoglobin, hematocrit, serum ferritin, and serum iron demonstrating iron deficiency anemia

	Results	Reference values with units
Hemoglobin	9.2 g/dL (Low)	13-18 g/dL
Hematocrit	31 % (Low)	38-50 %
WBC	5.33 x 10*3/uL (Normal)	4.3-10.0 x 10*3/uL
Platelet count	158 x 10*3/uL (Normal)	150-400 x 10*3/uL
Ferritin	4 ng/mL (Low)	20-230 ng/mL
Serum Iron	16 ug/mL (Low)	31-171 ug/mL
Serum Iron Binding Capacity	469 ug/mL (High)	250-460 ug/mL

**Figure 2:**
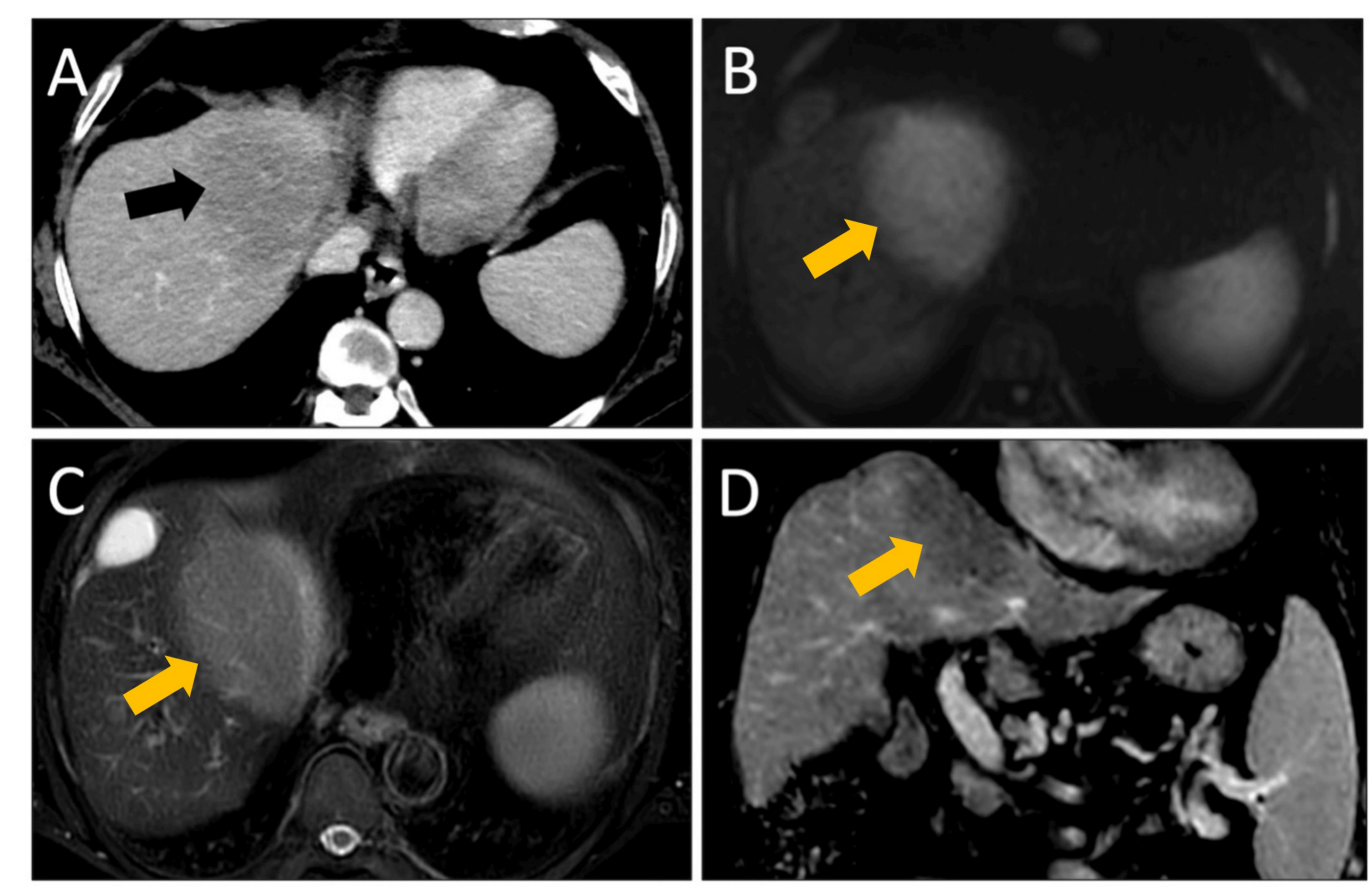
A) Axial CT scan with contrast of the upper abdomen with an ill-defined hypoenhancing subdiaphragmatic lesion (black arrow in A). B) Restricted diffusion on MRI diffusion-weighted imaging within the same lesion (yellow arrow in B). C) Axial T2-weighted MRI with a hyperintense lesion compared to background liver (yellow arrow in C). D) Coronal post-contrast T1-weighted imaging demonstrating subdiaphragmatic location of the hypoenhancing lesion (yellow arrow in D).

This was followed by a CT scan, which demonstrated a large, ill-defined subdiaphragmatic hepatic hypoenhancing lesion, not typical for hepatocellular carcinoma. MRI also demonstrated similar findings with a hypoenhancing area of restricted diffusion and T2 hyperintensity. Radiologically, this was diagnosed as a Liver Imaging Reporting and Data System (LI-RADS) category LR-M observation and likely represented a lymphoma (Figure 2).

**Figure 3:**
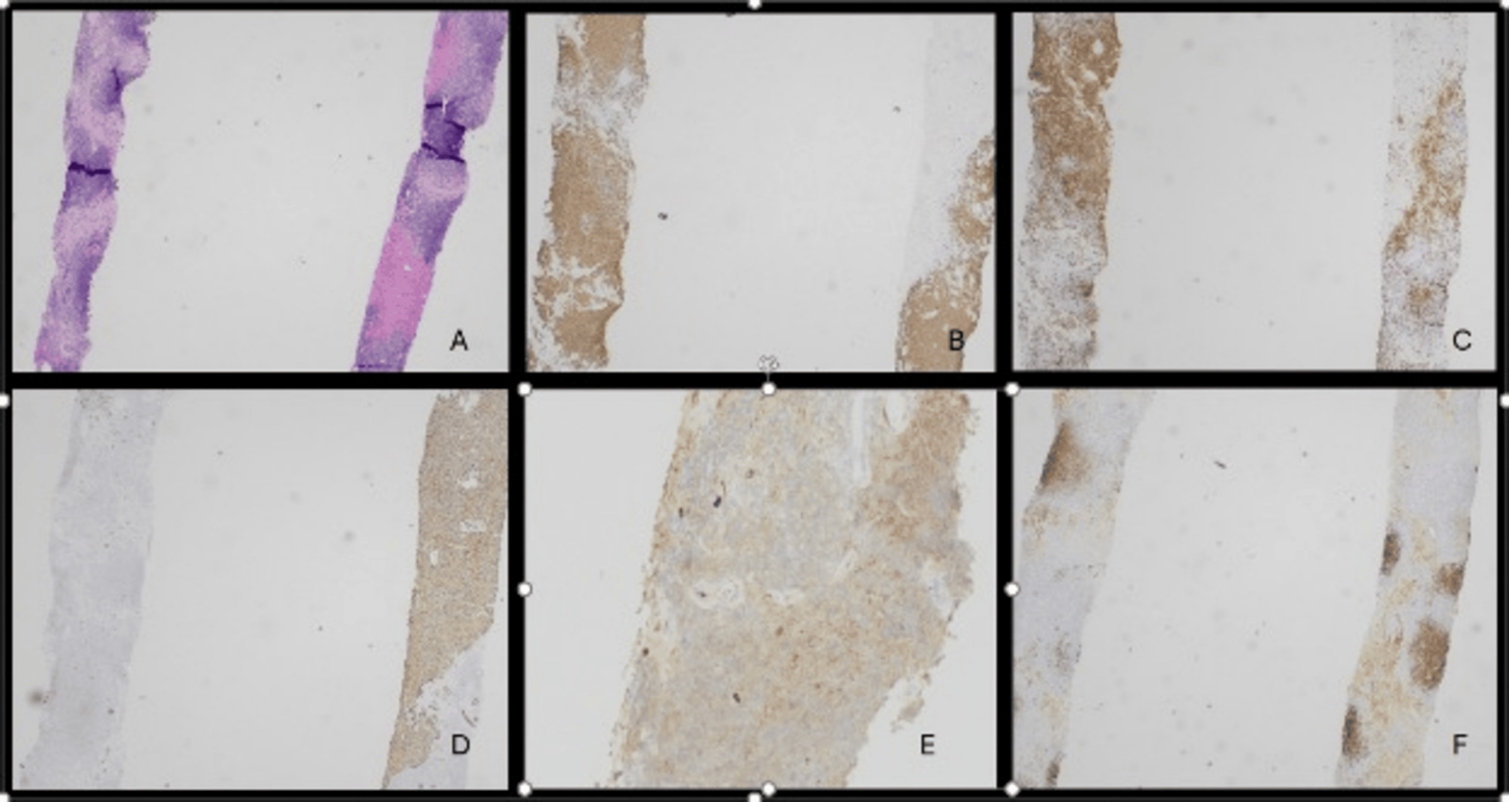
A) H&E stain of liver biopsy at 20x magnification showing liver parenchyma involved by a nodular and diffuse atypical lymphoid infiltrate in a few portal tracts and extending to the lobules. B) CD20 highlighting increased B cells in the liver. C) CD3 highlighting background T cells in the liver. D) CD138 highlighting increased focally clustered plasma cells in the liver. E) Kappa immunohistochemistry showing kappa restriction in atypical B cells. F) CD5 weakly positive in a subset of atypical B cells

**Figure 4:**
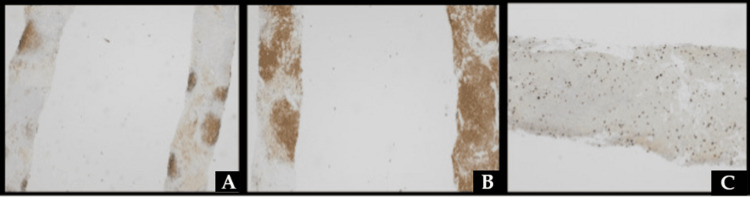
A) CD21 demonstrating preserved follicular dendritic meshwork. B) BCL2 positive in atypical B cells. C) Ki-67 demonstrating a low proliferative index of about 10% The atypical cells were negative for CD25, LEF-1, cyclin D1, and SOX11. BCL6 and CD10 showed patchy positivity in the germinal centers.

Ultrasound-guided biopsy was performed, and the patient was diagnosed with hepatic low-grade B-cell lymphoma with plasmacytic differentiation, likely marginal zone lymphoma based on lack of IgM and negative MYD88 (Figure 3 and Figure 4). Bone marrow biopsy was negative for lymphoma, and fluorodeoxyglucose (18F) positron emission tomography/computed tomography (18F-FDG PET-CT) did not reveal any additional lesions in the body.

**Figure 5:**
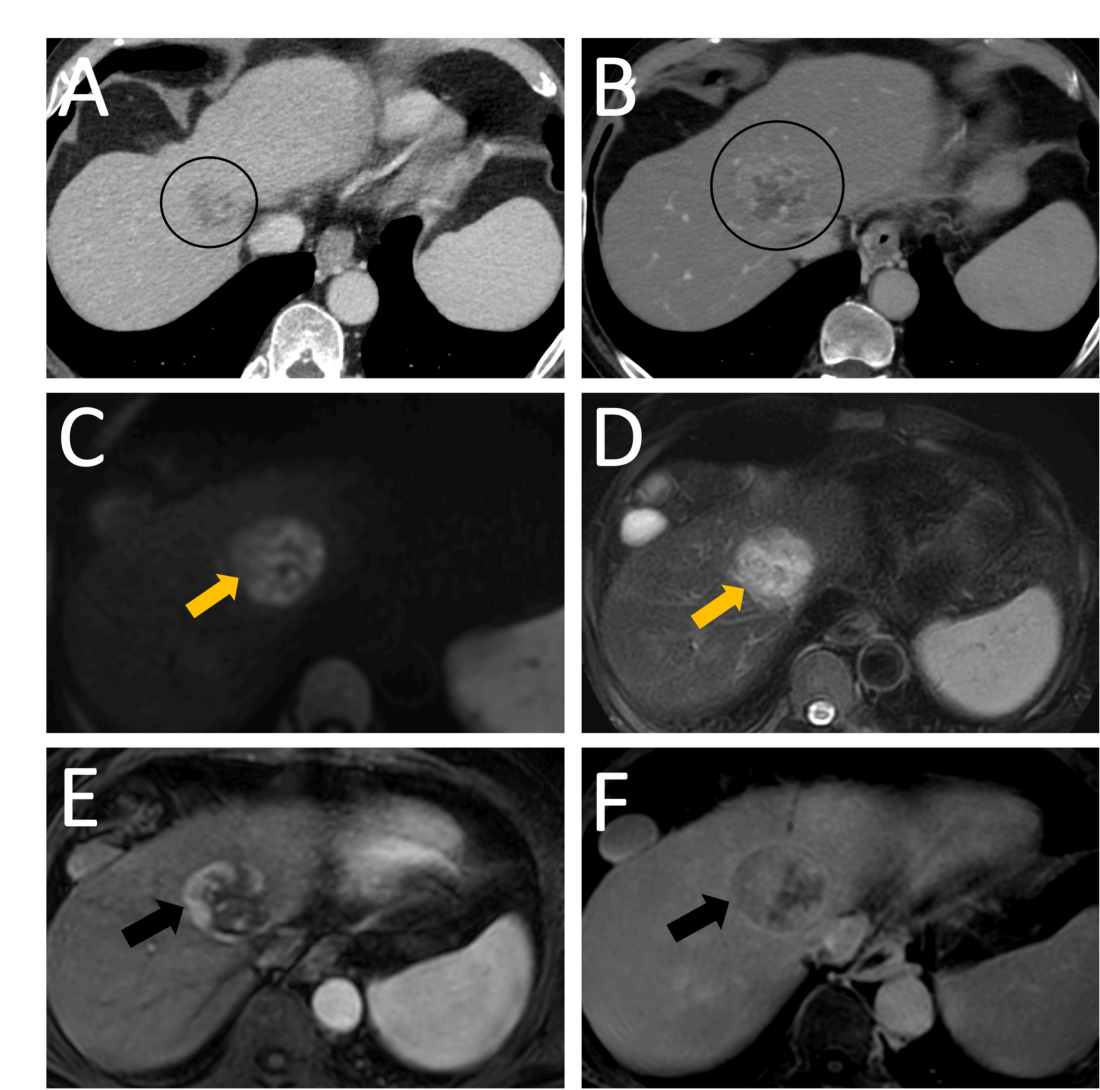
A) 6-month follow-up post-contrast axial CT scan with a decrease in the size of the hepatic lymphoma (black circle in A). B) 1-year follow-up post-contrast axial CT scan with an increase in the size of the lesion and a new heterogenous enhancement (black circle in B). C) Restricted diffusion on MRI diffusion-weighted imaging within the same lesion (yellow arrow in C). D) Axial T2-weighted MRI with hyperintense lesion compared to the background liver (yellow arrow in D). E) Axial T1-weighted post-contrast MRI in arterial phase with a non-rim hyperenhancement (black arrow in E). F) Axial T1-weighted delayed post-contrast MRI with washout and enhancing capsule (black arrow in F)

Treatment with BR (Bendamustine and Rituximab) with granulocyte-colony stimulating factor (G-CSF) and acyclovir prophylaxis was initiated, and the lymphoma initially decreased in size. However, on the one-year follow-up CT scan, this lesion increased in size, but with different imaging characteristics than those of a lymphoma. Other malignant etiologies or dedifferentiation to other tumor subtypes were considered. MRI of the liver demonstrated non-rim arterial phase hyperenhancement with washout and a capsule on delayed phase images, representing a LI-RADS category LR-5 observation, representing hepatocellular carcinoma (Figure 5).

**Figure 6:**
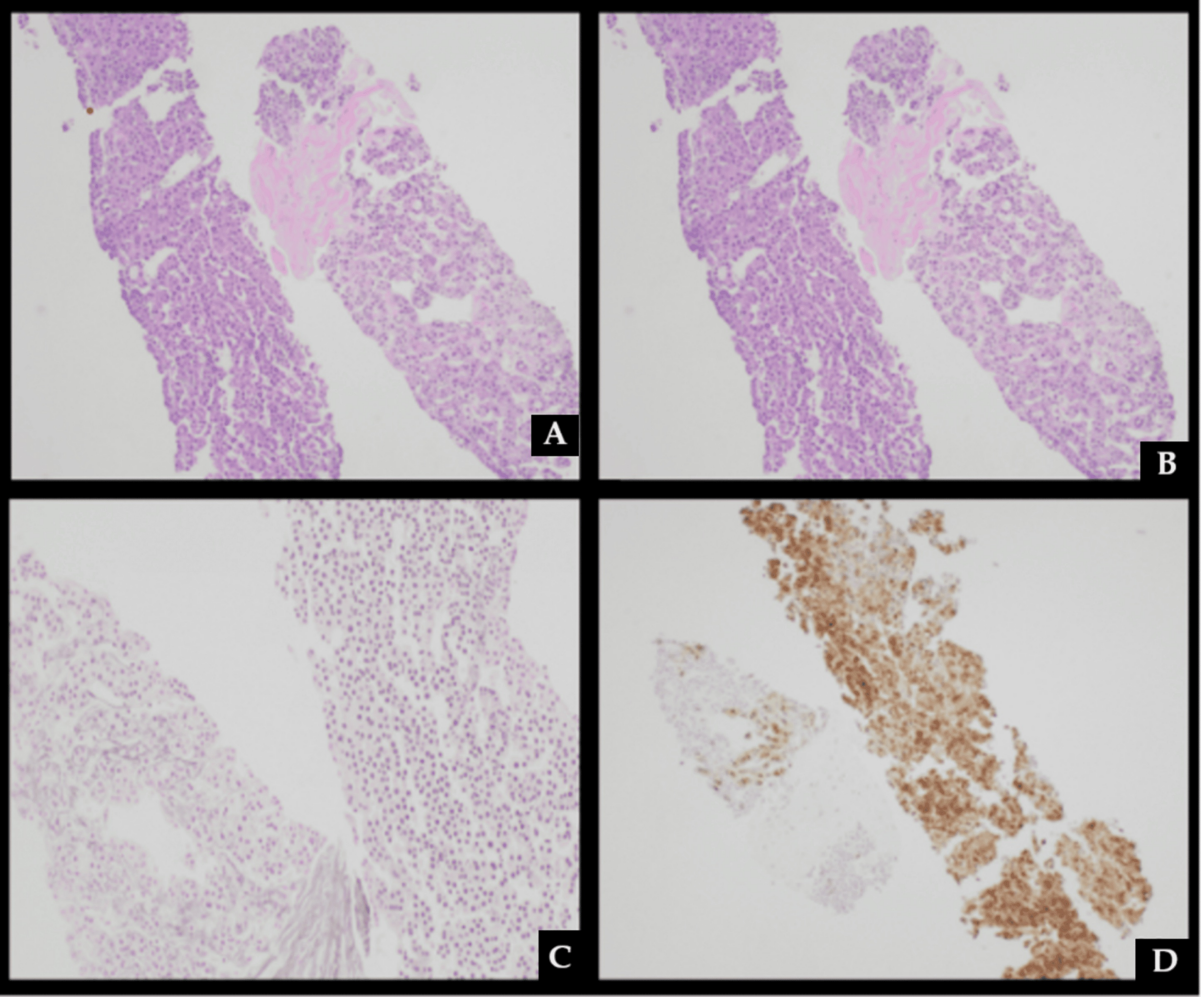
A and B) H&E stain of liver biopsy at 20x and 200x showing trabecular and pseudoacinar proliferation of atypical hepatocellular cells with rare unpaired vasculature and absent portal tracts. C) Reticulin stain demonstrating a disrupted reticulin meshwork. D) Glypican-3 demonstrating strong expression in the majority of carcinoma cells

A biopsy of this lesion was performed, which demonstrated a well-differentiated hepatocellular carcinoma, with flow cytometry negative for an abnormal B cell population (Figure 6). Unfortunately, he was not a transplant candidate given his age and agreed to proceed with Y-90 mapping and treatment under moderate sedation.

## Discussion

The development of hepatocellular carcinoma within a previously treated B-cell hepatic lymphoma is an extremely rare occurrence [5,6]. There have been only a handful of case reports in the literature describing the unique scenario of the co-occurrence of both PHL and hepatocellular carcinoma within the same patient. In these rare cases, patients had been previously diagnosed with B-cell hepatic lymphoma, which was subsequently treated, and during follow-up, they were found to have a new liver mass that was ultimately identified as hepatocellular carcinoma [2,4,5]. To our knowledge, this is the first case report describing the occurrence of hepatocellular carcinoma within a PHL, at the same, exact location, which can also be considered a transformation.

The exact mechanisms behind the development of hepatocellular carcinoma in this context remain unclear. However, it is believed that chronic liver disease, ongoing inflammation, and liver regeneration associated with both the primary B-cell hepatic lymphoma and hepatitis C infection may play a role in the onset of hepatocellular carcinoma [3,4]. Chronic infections with hepatitis C and hepatitis B viruses are well-established risk factors for both hepatocellular carcinoma and B-cell hepatic lymphoma [5]. A persistent viral infection, continuous liver damage, and subsequent regenerative processes create an environment that fosters the development of these cancers [2]. Additionally, the treatment for primary B-cell hepatic lymphoma, including chemotherapy or radiation therapy, can further damage the liver and promote regenerative processes, potentially contributing to the development of hepatocellular carcinoma.

During follow-up of any of the malignant lesions, a new imaging pattern on CT or MRI within the lesion of concern should prompt a biopsy with a histopathological diagnosis of a new tumor subtype or a new tumor altogether. Transformation of PHL into hepatocellular carcinoma can also be considered; however, there is no available literature, to our knowledge, to support this theory. Accurate diagnosis of hepatocellular carcinoma in a patient with a history of treated B-cell hepatic lymphoma can be challenging, as the imaging features of these two entities can overlap [1,5,7]. Nodular regenerative hyperplasia and large regenerative nodules, which are benign hepatic lesions, can also mimic the imaging appearance of hepatocellular carcinoma and should be considered in the differential diagnosis [8]. Cross-sectional imaging, notably an MRI scan, is the mainstay of diagnosis and lesion characterization. Although high-resolution 7T MRI imaging has been increasingly used for detailed evaluation of brain structures, its use in the abdomen is limited, with 1.5 T and 3T MRI remaining the most widely used imaging modalities alongside CT scans [9]. PHL appears as an ill-defined hypoenhancing lesion on CT and MRI. Newer PET imaging agents like fibroblast activation protein-based radio-tracers and routine 18F-FDG uptake can also help in the early detection of these malignancies and is highly beneficial for staging [10].

Histopathologically, PHL encompasses various subtypes, most commonly diffuse large B-cell lymphoma (DLBCL), followed by mucosa-associated lymphoid tissue lymphoma (MALToma). Diffuse large B-cell lymphoma is characterized by mitotically active, atypical large cells with pale cytoplasm, granular eosinophilic necrotic debris, and nuclei featuring condensed chromatin and prominent nucleoli. Immunohistochemical analysis confirms its diagnosis with markers such as CD20+ and pan T-cell markers like CD3 and Tdt. MALT lymphomas, by contrast, typically comprise small B cells, including marginal zone cells, monocytoid cells, and small lymphocytes, and may show plasma cell differentiation. Immunophenotypically, MALT lymphoma cells are CD20+, CD79a+, BCL2+, and IgM+. Negative staining for cyclin D1/SOX11 and CD10/BCL6 is useful for distinguishing MALT lymphoma from mantle cell lymphoma and follicular lymphoma, respectively. Coexpression of CD5, CD23, and LEF1 distinguishes chronic lymphocytic leukaemia from MALT lymphoma, although occasional expression of CD5 or CD23 may be seen in MALT lymphoma [11].

Hepatocellular carcinoma is characterized by the loss of normal hepatic architecture, including the absence of portal tracts, reduction or loss of the reticulin framework, and increased arterialisation with aberrant arterioles and sinusoidal capillarization. Hepatocellular carcinoma growth patterns include trabecular, solid (compact), pseudoglandular (pseudoacinar), and macrotrabecular configurations, with the latter associated with worse prognosis. A nodule-in-nodule growth pattern is a hallmark feature of HCC, and stromal invasion, defined by the absence of a CK7/CK19-positive ductular reaction around the nodule, is a reliable diagnostic indicator. Immunohistochemical staining, particularly with markers such as HSP70, glypican-3 (GPC3), and glutamine synthetase (GS), provides additional diagnostic specificity, with positivity for at least two markers strongly supporting the diagnosis of hepatocellular carcinoma. Despite advancements in diagnostic and therapeutic strategies, the prognosis for hepatocellular carcinoma remains poor, particularly in advanced cases, with a 5-year survival rate of less than 5% in symptomatic and unresectable hepatocellular carcinoma patients [12]. 

In such complex cases, a multidisciplinary approach involving hepatologists, oncologists, pathologists, and radiologists is crucial for establishing the correct diagnosis and guiding appropriate management.

## Conclusions

The development of primary hepatocellular carcinoma within a previously treated B-cell hepatic lymphoma in a hepatitis C-positive patient represents an extremely rare and complex clinical scenario. Although the co-occurrence of primary hepatic lymphoma and hepatocellular carcinoma within the liver has been described in a few case reports previously, this is the first instance of the two occurring at the same, exact location. Transformation of tumor type may be considered; however, there is no available literature to date to support this theory. The coexistence of these two malignancies is thought to be multifactorial, involving chronic liver disease, chronic inflammation, and liver regeneration processes associated with both the hepatitis C infection and the primary B-cell hepatic lymphoma, as well as potential treatment-related effects. Careful monitoring and surveillance for the development of hepatocellular carcinoma are essential in patients with a history of B-cell hepatic lymphoma, even after successful treatment. A change in radiological appearance should raise concern for a second malignancy, to trigger a repeat biopsy. Accurate diagnosis and appropriate management of this condition require a multidisciplinary approach, with close monitoring and prompt intervention to optimize patient outcomes. The prognosis for this unique clinical scenario is guarded, as the presence of underlying liver disease, multiple malignancies, and the potential for treatment-related complications can significantly impact the patient’s overall survival.
